# Identification of apoptosis-related microRNAs and their target genes in myocardial infarction post-transplantation with skeletal myoblasts

**DOI:** 10.1186/s12967-015-0603-0

**Published:** 2015-08-19

**Authors:** Qi Liu, Guo Qing Du, Zhi Tao Zhu, ChunYang Zhang, Xiao Wei Sun, Jing Jin Liu, Xia Li, Yong Shun Wang, Wen Juan Du

**Affiliations:** The Key Laboratory of Myocardial Ischemia, Chinese Ministry of Education, Harbin, China; Department of Cardiology, The Second Affiliated Hospital of Harbin Medical University, Harbin, 150086 China; Department of Ultrasound, The Second Affiliated Hospital of Harbin Medical University, Harbin, China; Department of Cardiac Surgery, The Second Affiliated Hospital of Harbin Medical University, Harbin, China

**Keywords:** microRNA, Skeletal myoblast, Myocardial infarction, Apoptosis, Gene expression

## Abstract

**Background:**

Skeletal myoblasts (SkMs) has provided a promising treatment for myocardial infarction (MI). Functioning as posttranscriptional regulators, microRNAs (miRNAs) play important roles in cardiac repairment and stem cell regulation. However, the correlation between miRNAs and their targeted genes in SkM cell therapy for MI was not fully understood.

**Methods:**

We explored the cardioprotection by SkMs in infracted rats and determined cardiac functions at 4 weeks. In addition, we compared the expression profiles of miRNAs and mRNAs in post-MI rats with or without SkM cell therapy using microarray. The concordance between miRNA expression and mRNA levels of potential target genes was confirmed by quantitative real-time PCR.

**Results:**

Quantitative echocardiography and histology showed improved cardiac function, attenuated heart infarcted area and inhibited cardiomyocyte apoptosis in the SkM group, compared with MI group. We identified that 160 miRNAs were differentially expressed in MI group as compared to the control group and 78 miRNAs were differentially expressed in the SkM treated group as compared to the untreated post-MI. We focused on a novel set of apoptosis-associated miRNAs and their target genes, among which 4 miRNAs (miR-30a-5p, miR-30c-5p, miR-145-5p, miR-140-3p), except one (miR-143-3p), were downregulated in the SkM treated group as compared to the untreated group. Furthermore, we found seven genes including Angptl4, Dpep1, Egr1, Eif5a, Tsc22d3, Irs2 and Cebpb that showed a linear correlation with which miRNAs.

**Conclusions:**

The downregulation of apoptosis-regulatory miRNAs and in turn upregulation of target genes may partially account for rescue effect of SKM therapy for MI.

**Electronic supplementary material:**

The online version of this article (doi:10.1186/s12967-015-0603-0) contains supplementary material, which is available to authorized users.

## Background

Myocardial infarction (MI) is a major cause of morbidity and mortality worldwide. In recent years, stem/progenitor cell therapies have been considered promising options to compensate for the loss of cardiomyocytes, which undergo apoptosis on a large scale. These apoptotic cell deaths account for acute cell loss in the infarct area and chronic cell loss in the infarct border zone [1, 2]. Recent reports have recognized skeletal myoblasts (SkMs) as potential candidates for stem cell therapy in treating MI because transplantation of SkMs for MI improved cardiac function recovery following ischemia in experimental animal studies and clinical trials [3]. However, the mechanisms of SkM-based therapy are not well understood.

miRNAs are 20–25 nucleotide noncoding RNAs that negatively modulate gene expression by acting posttranscriptionally to control the translation of messenger RNA (mRNAs) [4] through a binding site in the mRNA 3′ untranslated region (UTR). Several miRNAs have been identified as regulators of various physiological and pathological heart processes, including cardiac development, differentiation, arrhythmias, hypertrophy, remodeling, angiogenesis and MI [5–7]. Modulation of miRNA activity in the heart has been suggested to be an important mechanism that underlies the pathogenesis of cardiac diseases [8, 9]. Recent data have shown numerous miRNA dysregulations during MI, for example, MiR-210 and miR-1 were proven to improve cardiac function following MI by enhancing angiogenesis and inhibiting cardiomyocyte apoptosis [10, 11]; MiR-150 was shown to be downregulated in patients with acute myocardial infarction (AMI), atrial fibrillation, dilated cardiomyopathy and ischemic cardiomyopathy [12–14]; and overexpression of microRNA-99a attenuates heart remodeling and improves cardiac performance following myocardial infarction [15].

However, whether the molecular protected mechanisms of SkM-based therapy are involved in miRNA regulation is not clearly understood. Therefore, this study was designed to determine whether SkM-based therapies for MI involve a subset of miRNAs that regulate cardiomyocyte apoptosis. We found that intramyocardial delivery of SkMs into rat infracted hearts resulted in dramatic changes in some apoptosis-related miRNAs and expression of their predicted target mRNAs in MI tissue. The expression of the subset miRNAs and their predicted target mRNAs was confirmed by real-time PCR analysis. These results could provide a basis for the development of novel SkM-based therapeutics in MI.

## Methods

### Cell cultures

The isolation and purification of the primary SkMs were performed using the modified preplate method. The Wistar rats (1–3 days old) were sacrificed and soaked for 15 min in 75% ethanol. Approximately 1 g of muscle tissue was excised and minced, and was then digested in 3 ml 0.1% collagenase IA for 45 min and then 3 ml 0.25% trypsin for 5 min. Cell suspensions were centrifuged at 1,000 rpm for 10 min, and cell deposits were resuspended in the 3 ml DMEM. The cell suspension was purified with 60 min of preplating to eliminate fibroblasts. Purified myoblasts were cultured in growth medium containing DMEM supplemented with 20% fetal bovine serum at 37°C in humidified atmosphere with 5% CO_2_. SkMs were identified by immunostaining for desmin (1:100 Boster Bioengineering Co.) expression with DAPI for nucleus. The primary cells contained 90% desmin-positive SkMs (Data not shown).

### Surgical induction of MI and cell transplantation

All animal research protocols were approved by the Harbin Medical University’s Committee for the Care of Experimental Animals. Wistar rats (150–200 g) were anesthetized with 10% chloralhydrate (4 ml/kg, i.p.), and then mechanically ventilated with a small animal respirator. Following a left thoracotomy, the left anterior descending coronary artery was visualized, and a 7-0 ligature (CP Medical) was placed around the coronary artery. The rats were randomly divided into three groups: sham group: PBS is injected into Wild Type (WT) mice without coronary ligation; MI group: MI was created by permanently ligating the left anterior descending (LAD) coronary artery, then PBS is injected into the ischemic region of heart in WT mice; SkMs group means that SkMs cells were injected into WT mice with MI, before transplantation, SkMs were labelled using PKH26 cell tracker dye (2 × 10^6^ M, Sigma) for post-transplantation identification.

A total of 1.5 × 10^6^ cells were resolved in 100 μl medium and injected intramuscularly into four regions in the border zone distal to the ligation of the coronary artery immediately after induction of the MI. For Micro-array and miRNA analysis, we compared Sham vs MI, MI vs MI + SkMs and Sham vs MI + SkMs.

### Histology

Four weeks after surgery, the rats were euthanized, and their hearts were harvested and frozen in liquid nitrogen, and cryosectioned into 5 μm thick sections from the apex to the base. The surviving PKH26 positive SkMs were examined with a confocal laser-scanning microscope (Olympus Fluoview 1000, Olympus Corporation, Japan). To delineate fibrous tissues, paraffin sections were stained with Masson trichrome. Infarct size was defined as the sum of the infarcted epicardial and endocardial circumferences divided by the sum of the total LV epicardial and endocardial circumferences using computer-based planimetry. Quantitative assessment of each parameter was performed using image analysis software (Olympus BX41 + DP25). For apoptosis analysis, the sections were fixed in 4% paraformaldehyde and terminal deoxynucleotidyl transferase-mediated dUTP nick end-labeling (TUNEL) on different treatment groups was performed using an in situ Cell Death Detection Kit (Roche Applied Science, Penzberg, Germany) according to the manufacturer’s instruction protocol.

### Echocardiography

Cardiac function was assessed at 4 weeks post-MI by transthoracic echocardiography in n = 6 rats per group. Echocardiography was performed in the anesthetized rats using a Vivid 7 system (GE Healthcare, Milwaukee, WI, USA) equipped with a 10S transducer (8–12 MHz). LV parameters were obtained from two-dimensional images and M-mode interrogation in the long-axis view. LV fractional shortening (LVFS) and ejection fraction (LVEF) were measured.

### RNA isolation

Total RNA from rat heart samples was isolated using kits according to manufacturer’s instructions. For detecting the microRNA expression, miRcute miRNA Isolation Kit (TIANGEN BIOTECH (BEIJING CO, LTD), miRcute miRNA First-Strand cDNA Synthesis Kit and miRcute miRNA qPCR Detection Kit (SYBR Green) were used; for detecting mRNA, TRIzol RNA (Invitrogen), ThermoScript^TM^ RT-PCR System and a FastStart Universal SYBR Green Master (ROX) were used. RNA quality and quantity was measured by using Nanodrop spectrophotometer (ND-1000, Nanodrop Technologies) and RNA Integrity was determined by gel electrophoresis.

### microRNA arrays

microRNA expression analysis was performed by Kangchen (Kangchen, Bio-tech) using arrays based on the miRCURYTM LNA Array (v.18.0) (Exiqon) capable of detecting 567 miRNAs, according to array manual. In each group, three samples were mixed together for one microarray. Then the slides were scanned using the Axon GenePix 4000B microarray scanner (Axon Instruments, Foster City, CA, USA). The miRNA arrays in this experiment were performed as single color (Cy3). Scanned images were then imported into GenePix Pro 6.0 software (Axon) for grid alignment and data extraction. Replicated miRNAs were averaged and miRNAs that intensities ≥30 in all samples were chosen for calculating normalization factor. Expressed data were normalized using the Median normalization. After normalization, differentially expressed miRNAs were identified through Fold Change filtering. Hierarchical clustering was performed using cluster3.0 and treeview1.14. The microarray data was deposited to GEO (https://submit.ncbi.nlm.nih.gov/geo/submission/).

### mRNA arrays

mRNA expression analysis was performed by Kangchen (Kangchen, Bio-tech) using the Agilent DNA Microarray Scanner (part number G2505C) capable of detecting 28,000 probes. Agilent Feature Extraction software (version 11.0.1.1) was used to analyze acquired array images. Quantile normalization and subsequent data processing were performed with using the GeneSpring GX v11.5.1 software package (Agilent Technologies). After Quantile normalization of the raw data, genes that at least 3 out of 3 samples have flags in Detected (“All Targets Value”) were chosen for further data analysis. Differentially expressed genes were identified through Fold Change filtering. Hierarchical Clustering was performed using cluster3.0 and treeview1.14 GO analysis and Pathway analysis were performed by the Fisher’s exact test and test, and the threshold of significance was defined by *P* value and FDR.

### miRNA targets predictions

For rat, miRNA targets have been predicted using mirbase, miranda and mirdb and plugged within GeneSpring GX software in this study. The final results were predicted from the union part of the three databases.

### Quantitative real-time PCR analysis

For microRNA expression analysis, 1 μg of total RNA were extracted from heart tissues using miRcute miRNA Isolation Kit (TIANGEN BIOTECH (BEIJING) CO, LTD), then cDNA were synthesized using miRcute miRNA First-Strand cDNA Synthesis kit (TIANGEN BIOTECH (BEIJING) CO, LTD) as indicated in the manufacturer’s instructions. Synthesized cDNA samples were then subjected to RT-PCR using miRcute miRNA qPCR Detection Kit (SYBR Green) (TIANGEN BIOTECH (BEIJING) CO, LTD) according to instruction of the kit supplier, and the cDNA samples were not diluted. Each real-time PCR 20 μl reaction consisted of 2 μl of cDNA, 10 μl miRcute miRNA Premix, and 0.4 μl forward and 0.4 μl reverse primers. Reactions were carried out on CFX96TMReal-Time System (Bio-Rad). The primers sequence for miRNA-30a-5p, miRNA-30c-5p, miRNA-140-3p, miRNA-143-3p, miRNA-145-5p and small nuclear RNA U6 (U6) are given in Additional file 1: Table S1. The primers sequence for Angptl4, Dpep1, Egr1, Eif5a, Tsc22d3, Irs2, Cebpb and GAPDH are given in Additional file 2: Table S2. The threshold cycle (CT) is defined as the fractional cycle number at which the fluorescence passes the fixed threshold. The fold change expression of miRNAs among the third groups was examined by Bio-Rad CFX Manager quantitative PCR software. The fold change in expression of each gene was calculated using the 2^−∆∆CT^ method [16] with U6 and GAPDH as an internal control. The qRT-PCR reaction condition showed in Additional file 3: Table S3, and the Mastermix in the analysis of miRNAs and mRNAs was different, as showed in Additional file 3: Table S3. Samples were run in duplicate with RNA preparations from three independent experiments.

### Statistical analyses

The values were expressed as the mean ± SEM. Statistical analysis for these studies was determined using one-way ANOVA followed by least significance difference analysis using SPSS software. One-way ANOVA was used to analyzed cardiac function (LVEF,LVFS), Masson stain area analysis, apoptosis, microRNA and mRNA expression (by RT-PCR) between groups. The results were considered significant if *p* < 0.05. Statistical analysis for miRNA is described in the methodological section for each array.

## Results

### SkMs reduced the myocardial infarct area and improved cardiac function in rat hearts with MI

**Fig. 1:**
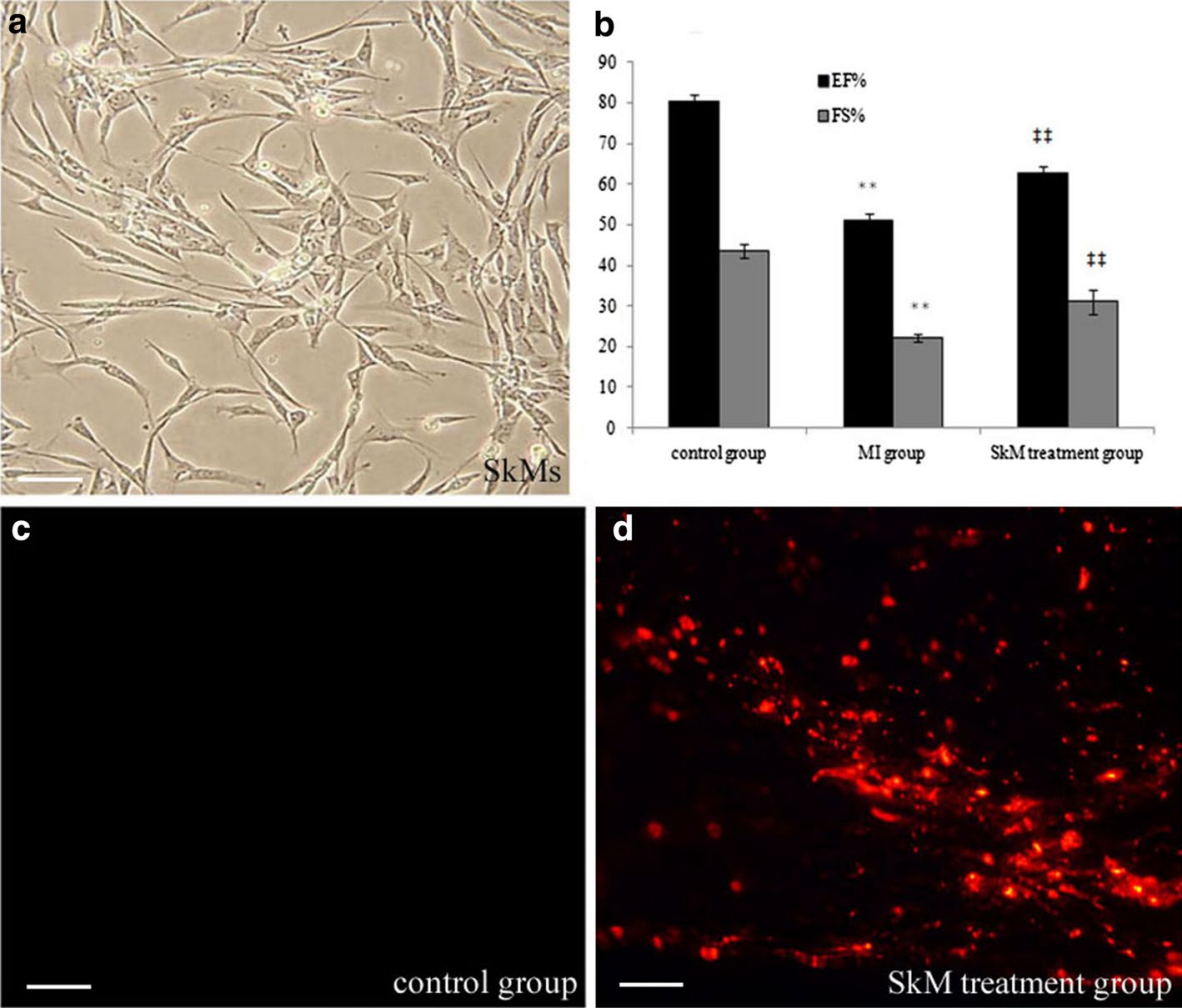
Effects of SkM cell therapy for rat MI on cardiac function. **a** Light-microscopy image of SkMs. *Scale bar* 50 μm. **b** Quantification of EF% and FS % were estimated at 4 weeks post-MI. The results are expressed as the mean ± SEM of six animals per group. **p < 0.01 vs. control group (Sham group); ^‡‡^ *p* < 0.01 vs. MI group. **c** There were no PKH26 + cells in the heart tissue of the control group. **d** Representative photomicrographs show donor PKH26 + transplanted cells in the border zone of a ischemic myocardium injected with SkMs as assessed under a confocal laser-scanning microscope. *Scale bars* 50 μm.

The beneficial effect of SkMs on MI was investigated using three randomized groups of experimental rats: a sham group, a MI group, and a SkM group. To evaluate the functional consequences of transplantation of SkMs in infarcted myocardium, echocardiography was performed in the three groups (n = 6 per group). MI rendered cardiac dysfunction, as indicated by reduced LVEF (51.03 ± 1.69% for the MI group vs. 80.38 ± 1.78% for the control group; *p* < 0.01) and LVFS (22.2 ± 0.95% for the MI group vs. 43.58 ± 1.64% for the control group; *p* < 0.01). Quantitative analyses showed that rats that received transplantation of SkMs following MI had a significantly better ejection fraction when compared to MI (64.67 ± 4.14% for the SkM group vs. 51.03 ± 1.69% for the MI group *p* < 0.01). Furthermore, SkM-transplanted rats also had significantly improved fractional shortening, post-MI, compared to the MI group (31.08 ± 2.98% for the SkM group vs. 22.2 ± 0.95% for the MI group *p* < 0.01). These data are shown in Fig. 1a, b.

**Fig. 2:**
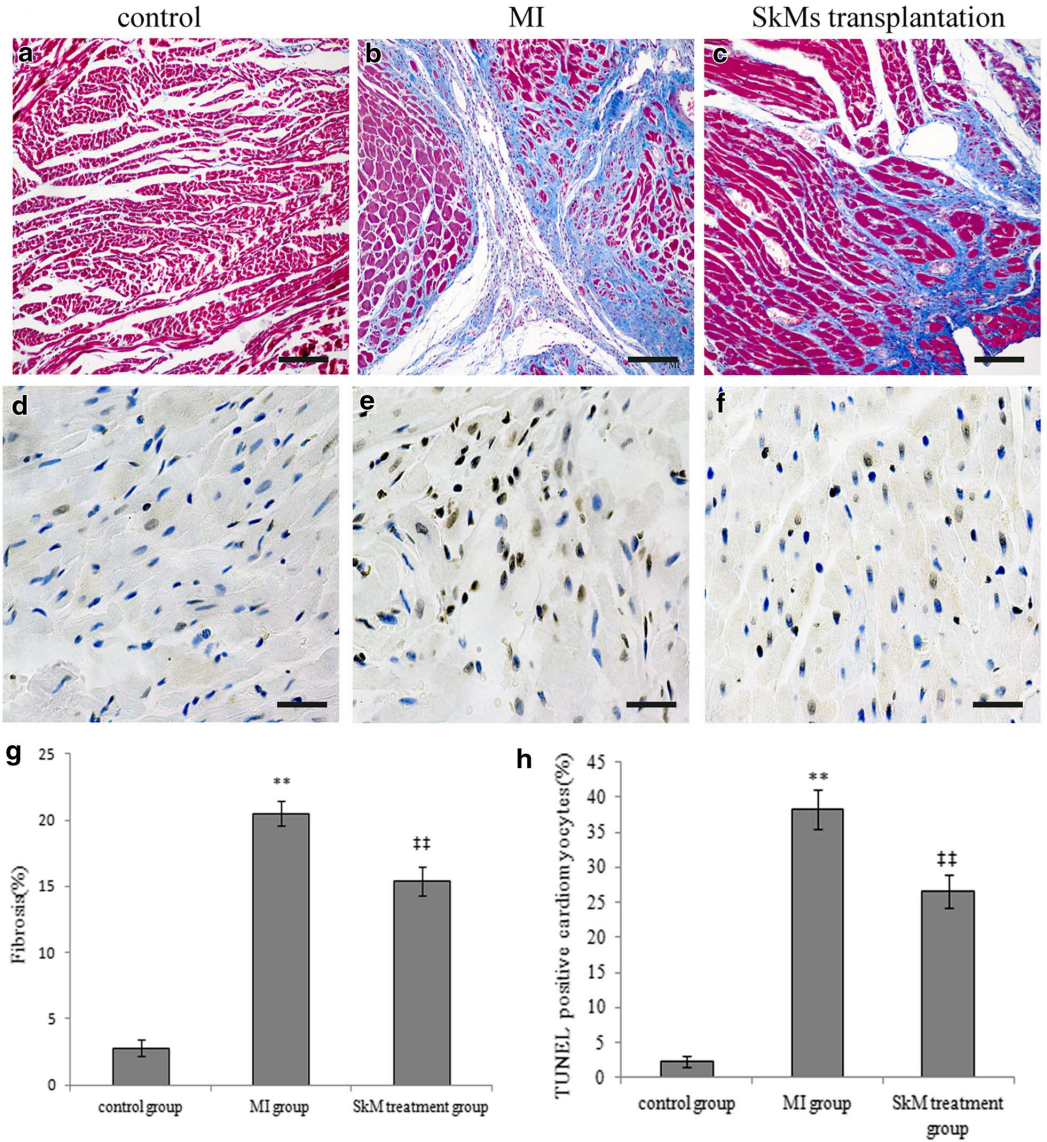
Infarct size and apoptosis were measured at 4 weeks post-MI. **a**–**c** Representative sections were stained with Masson trichome (collagen: *blue*, muscle: *red*) in different animal groups. *Scale bar* indicates 50 μm. **d**–**f** Representative sections were stained with TUNEL. *Scale bar* indicates 50 μm. **g** Quantitative data of fibrosis. Infarction size was attenuated in the SkM treatment group compared to the control group and MI group. **p < 0.01 vs. control group; ^‡‡^ *p* < 0.01 vs. MI group; n = 3 for each group. **h** The quantitative analysis of cardiomyocyte apoptosis was determined by the TUNEL staining assay. Data are presented as the mean ± SEM. Significance is indicated as **p < 0.01 vs. the control group; ^‡‡^ *p* < 0.01 vs. the MI group.

After imaging, animals were euthanized and hearts were explanted for histological analysis. Using a confocal laser-scanning microscope, we found a population of PKH26 positive cells in the explanted hearts of the SkM group and no PKH26 positive cells in the control groups (Fig. 1c, d). The heart infarction area in the MI group was associated with fibrosis, which was attenuated in the SkM group. At 4 weeks, Masson trichrome staining indicated a reduced infarction size in the SkM group compared to the MI group (Fig. 2a–c, g) (15.39 ± 1.04% for the SkM group vs. 20.52 ± 0.91% for the MI group *P* < 0.01). The rate of cardiomyocyte apoptosis was increased in MI rats compared to the sham group (38.25 ± 2.81% for the MI group vs. 2.25 ± 0.75% for the sham group; *p* < 0.01), whereas SkM transplantation substantially inhibited MI-induced cardiomyocyte apoptosis, as determined by TUNEL staining (26.59 ± 2.28% for the SkM group vs. 38.25 ± 2.81% for the MI group *p* < 0.01) (Fig. 2d–f, h).

### Global miRNA expression profiling in MI treated with SkMs from rat heart

The data showed a distinct miRNA expression signature (Additional file 4: Table S4). Compared to the sham group, the expression of 160 miRNAs significantly changed, with 60 miRNAs being down-regulated and 100 miRNAs being up-regulated at 4 weeks post-MI. In contrast, in the SkM group, differential expression of 78 miRNAs was observed at 4 weeks compared to the MI group, in which 46 miRNAs were down-regulated and 32 miRNAs were up-regulated. At 4 weeks, the expression of 164 miRNAs significantly changed in the SkM group compared to the sham group, and of the miRNAs, 66 miRNAs were down-regulated and 98 miRNAs were up-regulated. We also found that 68 miRNAs were reversed, thirteen miRNAs had reversal expression of downregulation, and 55 miRNAs showed reversal expression of upregulation after SkM treatment, in contrast to the sham group and MI group (Additional file 5: Table S5). It is therefore likely that these miRNA are functionally important in regulating SkM cell therapy for MI.

### Differentially expressed mRNA profiling in MI treated with rat heart SkMs

We analyzed mRNA arrays to compare changes in gene expression for the three groups. Similar to the miRNAs, the intersection of significantly up- or down-regulated mRNAs in the three groups’ tissue samples was determined using the Rat 4 × 44K Gene Expression Array. The expression of 1,627 genes was significantly altered between the MI group and the sham group at 4 weeks post-MI. Compared to the sham group, we found that 759 genes were significantly up-regulated, whereas the remaining 868 genes were significantly down-regulated in the MI group. In addition, the differential expression of 1,579 genes was observed at 4 weeks post-MI in the SkM group compared to the MI group, of which 632 were down-regulated and 947 were up-regulated (Additional file 6: Table S6). In contrast to the miRNA array data, there were more mRNAs altered (up-regulated or down-regulated) in the heart tissue samples.

### Analysis of significant trends in miRNA and mRNA expression

**Fig. 3:**
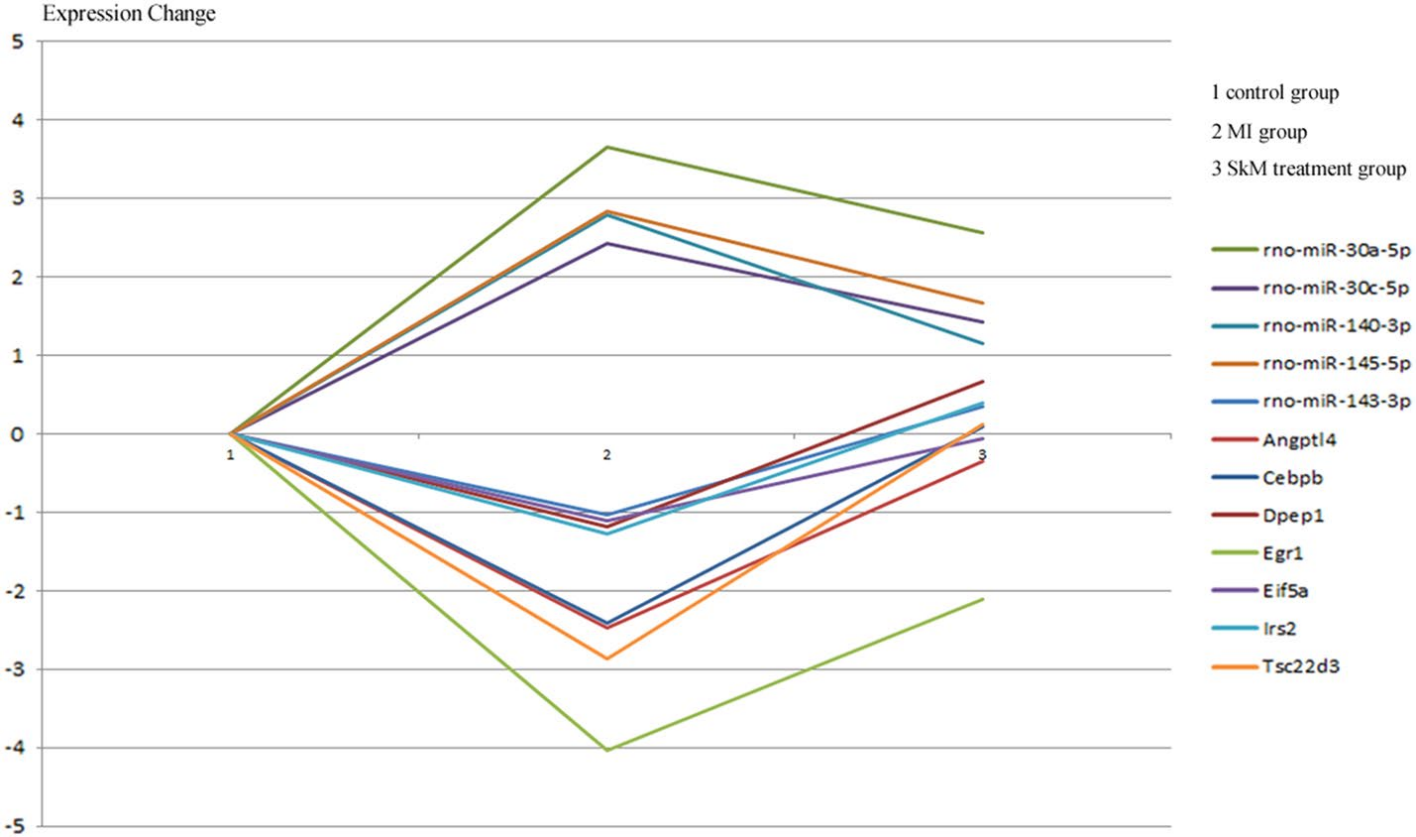
The expression trends of five key microRNAs and their seven targeted mRNAs in sham, MI and SkM treated groups. Fold change of each miRNA and target gene was normalized to sham group.

We observed a significant trend in the expression of some apoptosis-related miRNA and mRNA, including the expression of miR-30a-5p, miR-30c-5p, miR-145-5p, miR-143-3p, and miR-140-3p (Fig. 3). The horizontal axis represents different groups, and the vertical axis represents the logarithm of the ratio of miRNA and mRNAs expressing signal values to the control. When the *P* value was small, the impacts by the analysis on the trends of miRNA or mRNA expression were more remarkable.

### Identification of apoptosis-related miRNAs in rat MI’s treated with SkMs

**Fig. 4:**
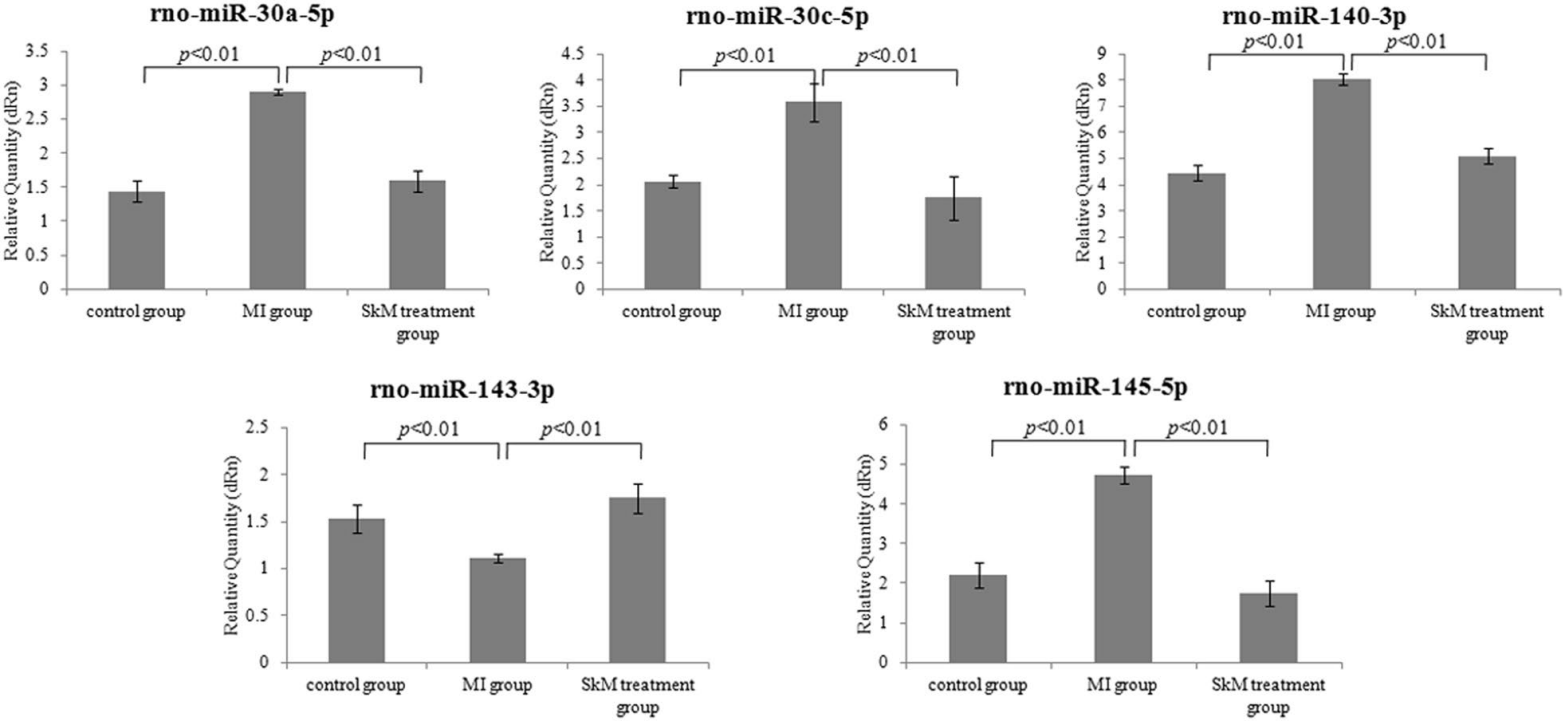
Analyses of the expression of rno-miR-30a-5p, rno-miR-30c-5p, rno-miR-140-3p, rno-miR-143-3p, and rno-miR-145-5p by RT-PCR. Data are presented as the Mean ± standard error of mean (SEM). Fold change was normalized to sham group.

To validate the miRNA array results, we used 2-cut off criteria and screened the microRNAs which were related to Wnt signal pathway and related to apoptosis. The expression of miR-30a-5p, miR-30c-5p, miR-145-5p, miR-143-3p, and miR-140-3p in the infarcted border zone region was measured by real-time analysis 4 weeks after MI. Interestingly, as shown in Fig. 3, at 4 weeks post-MI, with the exception of miR-143-3p, changes in miR-30a-5p, miR-30c-5p, miR-145-5p, and miR-140-3p expression tended to increase compared to the control group, whereas the expression of these four miRNAs decreased and the expression of miR-143-3p increased in the SkM +MI group, suggesting that these miRNAs may be associated with SkM therapy in myocardial injury. The results from real-time qPCR analysis showed high concordance with our microarray results for all investigated transcripts, as shown in Fig. 4.

### Pathway analysis and GO analysis of targeted mRNA changes

In total, among the target genes, KEGG pathways were overrepresented to help us obtain useful information about the function of these targets, as shown in Additional file 7: Figure S1. Several genes implicated in MI are involved in adrenergic signaling in cardiomyocytes, the TNF signaling pathway, the TGF-beta signaling pathway, and metabolic pathways.

To further explore the relationship between miRNAs and gene function in the three groups at 4 weeks, we built a miRNA-GO-network in heart tissue. The five key miRNAs in the network were identified as miR-30a-5p, miR-30c-5p, miR-145-5p, miR-143-3p, and miR-140-3p. GO biological processes were also observed to be associated with up- and down-regulated genes, respectively, as shown in Additional file 8: Figure S2 and Additional file 9: Figure S3. Genes involved in positive regulation of cell death, response to hypoxia, and negative regulation of apoptotic process were found, as shown in Additional file 10: Table S7.

### Differential expression of mRNAs targeted by regulated miRNAs post-MI

To examine the potential mRNA targets of regulated miRNAs, mRNA and miRNA array data sets were analyzed using a microRNA and mRNA integrated analysis software that integrates mRNA and miRNA expression data based on miRNA-predicted targets (Additional file 11: Table S8). We constructed a miRNA-Gene-network of these differential genes and miRNAs in heart tissue. The five key miRNAs in the network were identified as miR-30a-5p, miR-30c-5p, miR-145-5p, miR-143-3p, and miR-140-3p, as shown in Additional file 12: Figure S4.

### miRNA target prediction and validation

**Fig. 5:**
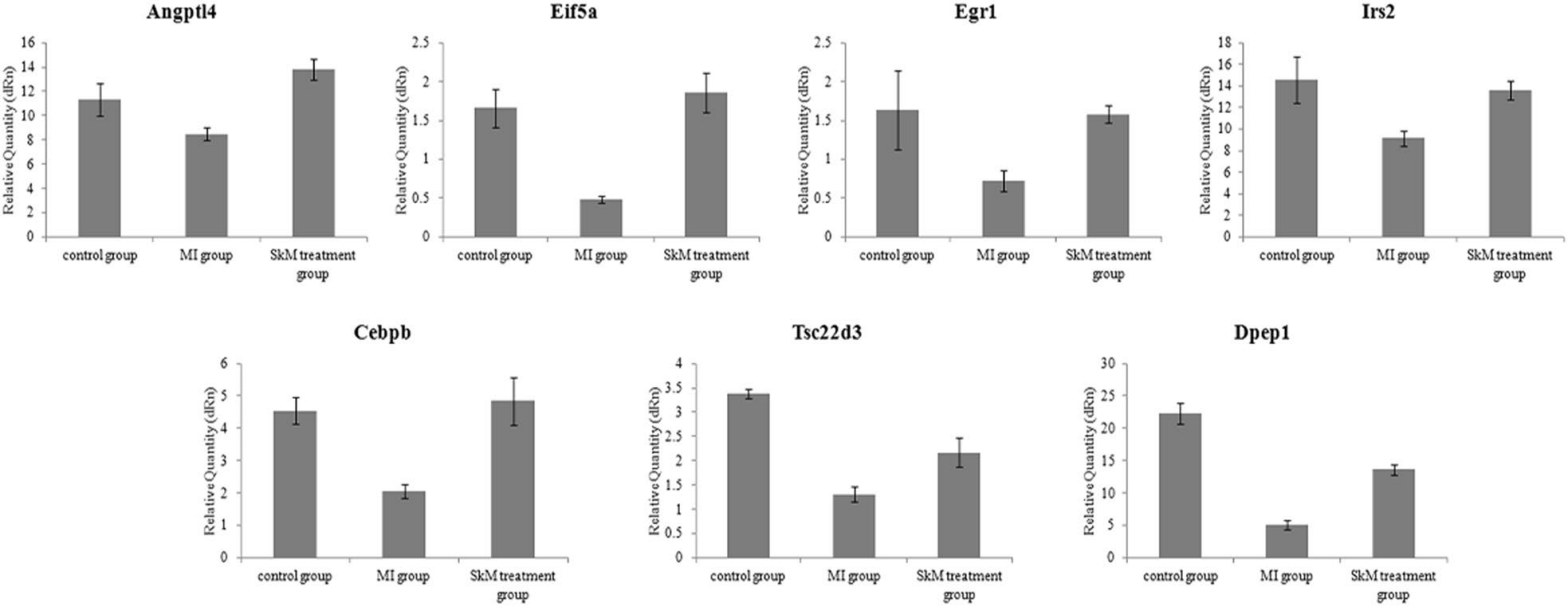
Expression of a subset of target genes differentially regulated in the array analysis was analyzed by RT-PCR. Gene expression in the three groups was compared. The diagrams show the relative mRNA levels.

MIRANDA, MICROCOSM, and MIRDB programs were employed to predict potential targets of five key miRNAs, miR-30a-5p, miR-30c-5p, miR-145-5p, miR-143-3p, and miR-140-3p. The targets of each miRNA predicted by three different programs were graphed in Venn’s diagram as shown in Additional file 13: Figure S5, among which seven anti-apoptotic genes: Angpt14, Eif5a, Egr1, Irs2, Cebpb, Tsc22d3, and Dpep1 were selected out for further validation. qRT-PCR results showed that the expression of the above seven target genes was negatively correlated to the levels of five miRNAs we identified (Fig. 5). Furthermore, qPCR validation results showed high concordance with our microarray results for all investigated transcripts, as shown in Fig. 5.

## Discussion

Our study is the first example of simultaneous miRNA/mRNA expression profiling of MI heart tissue treated by SkM transplantation to identify novel target genes of apoptosis-related microRNAs. We focused on changes in some apoptosis-associated miRNA and in gene expression in response to SkM MI therapy, corroborated some of the results obtained from microarrays with real-time qPCR analysis, and selected some significant miRNAs, including miR-30a-5p, miR-30c-5p, miR-145-5p, miR-143-3p, and miR-140-3p, which were involved with anti-apoptotic target genes such as Angptl4, Dpep1, Egr1, Eif5a, Tsc22d3, Irs2 and Cebpb. Accumulating research has shown that miRNAs play fundamental roles in cardiac development and cardiovascular disease [17, 18]. Several studies in human and animal model samples (not only blood samples but also human MI tissue samples) have reported differential miRNA expression in response to MI acutely (animal) or chronically (human) [19–22]. Our data also suggest that some apoptosis-associated miRNAs, such as miR-30a-5p, miR-30c-5p, miR-145-5p, miR-143-3p, miR-140-3p and their target genes, may play an important role in myocardial injury after MI.

In this study, we found that treatment with SkMs improved cardiac function of rat hearts post-MI. Quantitative echocardiography analyses showed that rats that received SkMs transplantation following MI had a significantly improved ejection fraction when compared to the MI group. Furthermore, MI rats transplanted with SkMs also had significantly improved fractional shortening compared to the MI group. By histology, the heart infarction area in the MI group was associated with fibrosis, which was attenuated by SkM transplantation, and SkM transplantation substantially inhibited MI-induced cardiomyocyte apoptosis, as determined by TUNEL staining. We also found that 68 miRNAs were reversed, thirteen miRNAs had reversal expression of downregulation, and 55 miRNAs showed reversal expression of upregulation after SkM treatment, in contrast to the sham group and MI group (Additional file 5: Table S5). It is therefore likely that these miRNAs are functionally important in the regulation of SkM cell therapy for MI. The cardioprotective effects of SkM transplantation included improved cardiac function and lower infraction size due to cardiomyocyte apoptosis, which was consistent with previously published experimental and clinical studies [23].

In this experiment, we focused on a subset of apoptosis-related miRNAs, including miR-30a-5p, miR-30c-5p, miR-145-5p, miR-143-3p, and miR-140-3p, and mRNAs involved in anti-apoptotic target genes such as Angptl4, Dpep1, Egr1, Eif5a, Tsc22d3, Irs2 and Cebpb. With the exception of miR-143-3p, we observed that changes in the expression of miR-30a-5p, miR-30c-5p, miR-145-5p, and miR-140-3p at 4 weeks post-MI tended to increase compared to the control group, whereas expression of these four miRNAs decreased and the expression of miR-143-3p increased in the SkM treatment group, suggesting that these miRNAs could be associated with the SkM therapy of myocardial injury. Some researchers ever reported that miR-21 and miR-181 were significantly upregulated post-MI [24, 25], which is consistent with our microRNA assay data. However, we noticed that unlike the five key miRs we identified neither miR-21 nor miR-181 significantly changed upon SkM treatment post-MI in our study. Such inconsistence might be due to different experiment conditions (e.g. time for tissue harvest).

Accumulating evidence strongly suggests that the miR-145 family has a relationship with apoptosis [26–28]. In our study, the expression of miR-145-5p increased after MI and declined after SkM treatment. This result was consistent with a human study in MI patients showing that the concentration of circulating miR-145 correlates with that of cTnI and CK-MB; increased circulating miR-145, cTnI and CK-MB are associated with worse outcomes in human MI blood samples [29]. We also demonstrated that miR-140, miR-143 and miR-145 might also collectively cooperate in MI treatment with SkMs by regulating targeted genes such as Egr1. A study demonstrated that knockdown of miR-140 was able to reduce myocardial infarct sizes in an animal model. They also observed that miR-140 could suppress the expression of Mfn1 and that it exerted its effect on mitochondrial fission and apoptosis through Mfn1 targeting [30]. In this study, we selected four apoptosis-related genes to be assayed by RT-PCR, including Angptl4, Dpep1, Eif5a, and Tsc22d3, which are regulated by miR-140-3p. MiR-143 and miR-145 were direct transcriptional targets of serum response factor (SRF), myocardin and Nkx2.5 and were down-regulated in injured or atherosclerotic vessels containing proliferating, less differentiated smooth muscle cells. miR-143 and miR-145 act as integral components of the regulatory network whereby SRF controls cytoskeletal remodeling and phenotypic switching of smooth muscle cells (SMCs) during vascular disease [31].

MiR-30 was shown to induce apoptosis [32] and regulate cell motility by influencing the extracellular matrix remodeling process [33–35]. We here confirm that miR-30a-5p and miR-30c-5p might target genes participating in the regulation of apoptosis. In this study, we found that, for miR-30a-5p at 4 weeks, 26 target genes were down-regulated and 10 target genes were up-regulated in the MI group compared to the control group. Twenty-seven target genes were up-regulated and 9 three target genes were down-regulated in the SkM treatment group compared with the MI group. For miR-30c-5p, 25 target genes were down-regulated and 10 target genes were up-regulated at 4 weeks in the MI group compared with the control group. Twenty-seven target genes were up-regulated and 8 three target genes were down-regulated in the SkM treatment group compared to the MI group. Moreover, most targeted genes were cooperated on by miR-30a-5p and miR-30c-5p. We observed that, together, miR-30a-5p and miR-30c-5p might target genes involved in the regulation of apoptosis in MI treated with SkMs. Moreover, we confirmed that these miRNAs might play an important role in MI by regulating targeted genes such as Cebpb and Irs2, which were detected by RT-PCR.

## Conclusion

**Fig. 6:**
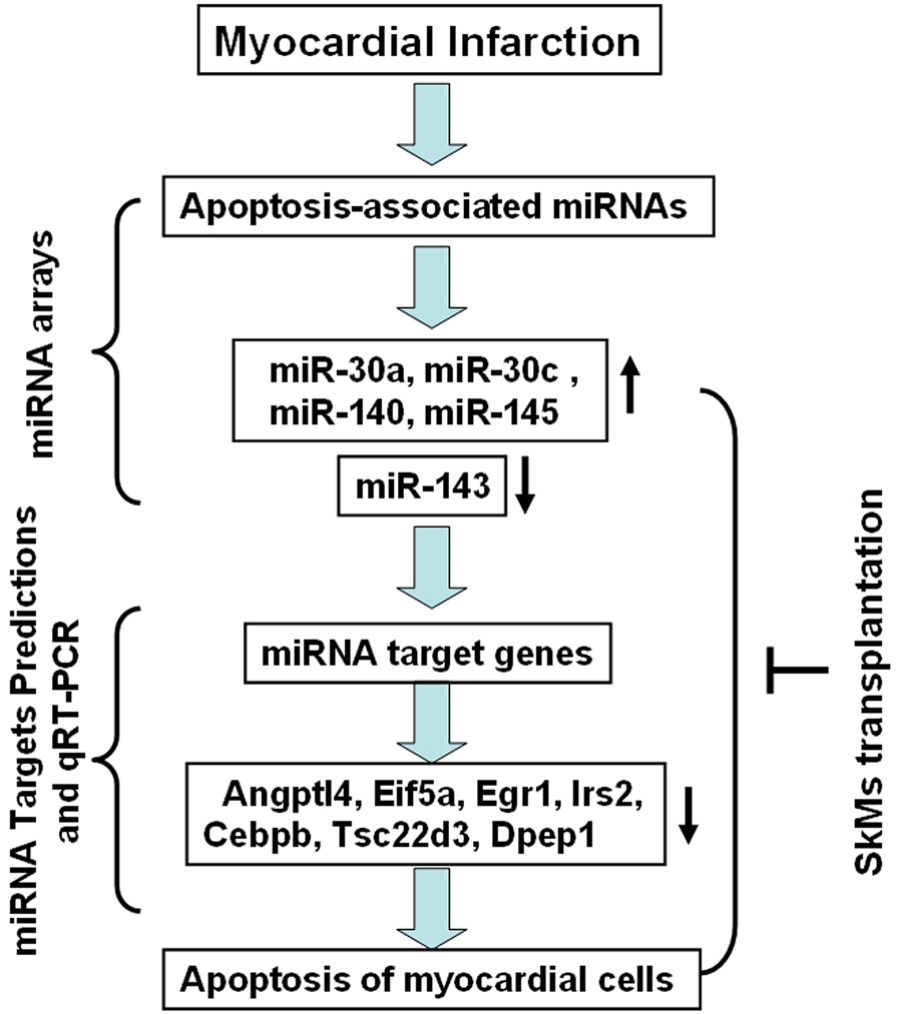
Diagram for identifying apoptosis-associated miRNAs and their target genes in myocardial infarction. Total RNA was isolated from infracted cardiac tissues for miRNA array. Up- or down-regulated apoptosis-associated miRNAs were indicated in myocardial infarction. Potential target genes regulated by apoptosis-associated mRNAs were screened out and further validated by qRT-PCR. The inhibitory effect on the change of apoptosis-associated miRNAs was observed after skeletal myoblast transplantation therapy.

 this is the first report to perform a global comparative study of miRNA and mRNA expression in rat MI heart tissues treated with SkM transplantation. We identified that SkMs improved cardiac function and attenuated the infracted area of the MI heart. We focused on five apoptosis-related miRNAs (miR-30a-5p, miR-30c-5p, miR-145-5p, miR-143-3p, and miR-140-3p), demonstrated their changes after SkMs treatment and filtered out 7 anti-apoptotic target genes, namely, Angpt14, Eif5a, Egr1, Irs2, Cebpb, Tsc22d3, and Dpep1, in the heart tissues (Fig. 6). Our results provide an excellent starting point for further studies regarding the functional properties of differentially expressed miRNAs in the context of SkM transplantation and the development of miR-based stem cell therapeutics for MI and demonstrated that target genes could become new therapeutic targets for heart recovery after MI.

## Additional files

Additional file 1:
**Table S1.** Primers of miR-30a-5p, miR-30c-5p, miR-145-5p, miR-143-3p, and miR-140-3p used in qRT-PCR. Additional file 2:
**Table S2.** Primers of Angpt14, Eif5a, Egr1, Irs2, Cebpb, Tsc22d3, and Dpep1 used in qRT-PCR. Additional file 3:
**Table S3.** qRT-PCR reaction system for microRNA and mRNA. Additional file 4:
**Table S4.** Primary data of microRNA arrays in rat hart simples. 1, Sham group; 2, MI group; 3, SkM group. Additional file 5:
**Table S5.** Restoring microRNA data of microRNA arrays in rat hart simples. Additional file 6:
**Table S6.** Primary data of mRNA arrays in rat hart simples. 1, Sham group; 2, MI group; 3, SkM group Additional file 7:
**Figure S1.** Pathway analysis based on miRNA targeted genes showed significant pathways targeted by rno-miR-30a-5p, rno-miR-30c-5p, rno-miR-140-3p, rno-miR-143-3p, and rno-miR-145-5p. The vertical axis is the pathway category, and the horizontal axis is the enrichment of pathways. Additional file 8:
**Figure S2.** Venn’s diagram for target genes of rno-miR-30a-5p, rno-miR-30c-5p, rno-miR-140-3p, rno-miR-143-3p, and rno-miR-145-5p predicted by MIRANDA, MICROCOSM, MIRDB. Additional file 9:
**Figure S3.** GO-Analysis based on miRNA targeted genes showed significant function of target genes by rno-miR-30a-5p, rno-miR-30c-5p, rno-miR-140-3p, rno-miR-143-3p, and rno-miR-145-5p. The vertical axis is the significant functions, and the horizontal axis is the enrichment of functions. Additional file 10:
**Table S7.** Genes involved in positive regulation of cell death, response to hypoxia, and negative regulation of apoptotic process. Additional file 11:
**Table S8.** Primary data of miRNA-predicted target arrays in rat hart simples. Additional file 12:
**Figure S4.** MicroRNA-GO-Network, screening out the main function of the target genes regulated by rno-miR-30a-5p, rno-miR-30c-5p, rno-miR-140-3p, rno-miR-143-3p, and rno-miR-145-5p. The red nodes represent the microRNAs, and the blue nodes represent functions of the target genes. Additional file 13:
**Figure S5.** Building the microRNA-Gene-Network by using the regulatory relationships between microRNAs and target genes. The red nodes represent the microRNAs, and the blue nodes represent the target genes.
