# Supplementary material for: Identification of apoptosis-related microRNAs and their target genes in myocardial infarction post-transplantation with skeletal myoblasts
Source: J Transl Med. 2015 Aug 19;13:270. doi: 10.1186/s12967-015-0603-0 (PMC4539916; doi:10.1186/s12967-015-0603-0)
Supplement: Additional file 1: — Table S1. Primers of miR-30a-5p, miR-30c-5p, miR-145-5p, miR-143-3p, and miR-140-3p used in qRT-PCR. [file 12967_2015_603_MOESM1_ESM.docx]

| Gene | Annealing temperature (℃) | primer sequences |
| --- | --- | --- |
| rno-miR-140-3p | 57℃ | GTACCACAGGGTAGAACCACGGA |
| rno-miR -143-3p | 58℃ | CGTGAGATGAAGCACTGTAGCTCA |
| rno-miR -145-5p | 59℃ | TCCAGTTTTCCCAGGAATCCCT |
| rno-miR -30a-5p | 58℃ | GATGTAAACATCCTCGACTGGAAG |
| rno-miR -30c-5p | 57℃ | F:5’CCAGCGTGTGTAAACATCC3’  R:5GTCGTATCCAGTGCAGGGTCCGAGGTATTCGCACTGGATACGACGCTGAG3’ |
| U6 | 57℃ | F:5’GCTTCGGCAGCACATATACTAAAAT3’   R:5’CGCTTCACGAATTTGCGTGTCAT3’ |

Supplemental table 1. Primers used in qRT-PCR
