# Supplementary material for: Identification of apoptosis-related microRNAs and their target genes in myocardial infarction post-transplantation with skeletal myoblasts
Source: J Transl Med. 2015 Aug 19;13:270. doi: 10.1186/s12967-015-0603-0 (PMC4539916; doi:10.1186/s12967-015-0603-0)
Supplement: Additional file 2: — Table S2. Primers of Angpt14, Eif5a, Egr1, Irs2, Cebpb, Tsc22d3, and Dpep1 used in qRT-PCR. [file 12967_2015_603_MOESM2_ESM.docx]

| Gene | Annealing temperature (℃) | primer sequences |
| --- | --- | --- |
| Angptl4 | 52℃ | F 5’  TGGGGTAGACAAGACTTCG  3’  R 5’  GTCCACAGAGCCGTTCAG  3’ |
| Dpep1 | 54.4℃ | F 5’  AGTCTGATTGGCGTGGAAG  3’  R 5’  GCAGTTGTGCGTAAGGGTC  3’ |
| Egr1 | 54.4℃ | F 5’  GACAAGTTATCCCAGCCAAAC  3’  R 5’  GCAGAGGAAGACGATGAAGC  3’ |
| Eif5a | 52℃ | F 5’  GCAGTGCTCAGCATTACG  3’  R 5’  CCTTTTGATGTTGGGGAC  3’ |
| Tsc22d3 | 54.4℃ | F 5’  GGTGGCGGTCTATCAACTG  3’  R 5’  GCTCACGAATCTGCTCCTTT  3’ |
| Irs2 | 54.4℃ | F 5' CCTGTGGGTCGGATTTTG 3'  R5' CTCTTGGGCTCAGTGGGTAG3' |
| Cebpb | 52℃ | F 5' GCGCCATCGACTTCAG 3'  R 5'CGGACGGCTTCTTGCT 3' |
| GAPDH | 54.4℃ | 5’-GGCACAGTCAAGGCTGAGAATG-3’  5’-ATGGTGTGTAAG ACGCCAGTA-3’ |

Supplemental table 2. Primers used in qRT-PCR
