# Supplementary material for: Identification of apoptosis-related microRNAs and their target genes in myocardial infarction post-transplantation with skeletal myoblasts
Source: J Transl Med. 2015 Aug 19;13:270. doi: 10.1186/s12967-015-0603-0 (PMC4539916; doi:10.1186/s12967-015-0603-0)
Supplement: Additional file 3: — Table S3. qRT-PCR reaction system for microRNA and mRNA. [file 12967_2015_603_MOESM3_ESM.doc]

(1) miRNAs real-timePCR reaction system：

| 2×miRute miRNA Premix | 5.0µl |
| --- | --- |
| Forward Primer | 0.2µl |
| Reverse Primer | 0.2µl |
| cDNA | 1.0µl |
| ddH_2_O | 3.6µl |

***(2) mRNAs real-time PCR reaction system：***

| Reagent | Volume |
| --- | --- |
| FastStart Universal SYBR Green Master(ROX) | 5ul |
| Forward primer(30um) | 0.5ul |
| Reverse primer(30um) | 0.5ul |
| Water PCR-grade | 3ul |
| cDNA | 1ul |
| Total volume | 10ul |

*(3)* ***qPCR reaction condition:***

95.0 ℃ for 10:00

95.0 ℃ for 0:10

54.4 ℃ for 0:30 （Dpep1，Egr1，Tsc22d3，Irs2，GAPDH）

52.0 ℃ for 0:30(Angptl4, Eif5a,Cebpb)

Plate read

72.0 ℃ for 0:15

Go to 2 step. 45 more times

Melt Curve 65℃ to 95℃; Increment 0.5℃ for 0:05
