# Supplementary figures and images for: Identification of apoptosis-related microRNAs and their target genes in myocardial infarction post-transplantation with skeletal myoblasts
Source: J Transl Med. 2015 Aug 19;13:270. doi: 10.1186/s12967-015-0603-0 (PMC4539916; doi:10.1186/s12967-015-0603-0)

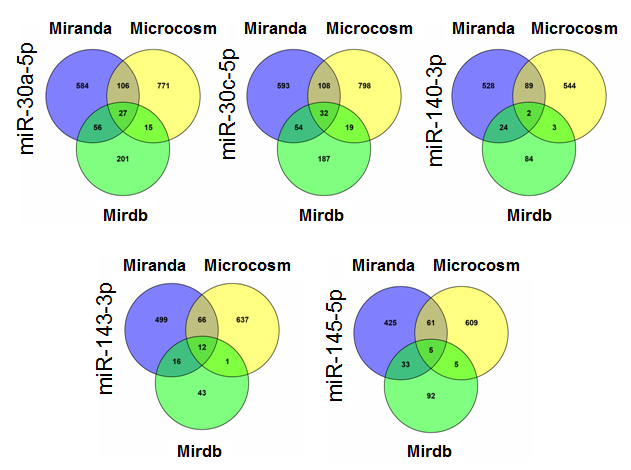

Supplement: Additional file 7: — Figure S1. Pathway analysis based on miRNA targeted genes showed significant pathways targeted by rno-miR-30a-5p, rno-miR-30c-5p, rno-miR-140-3p, rno-miR-143-3p, and rno-miR-145-5p. The vertical axis is the pathway category, and the horizontal axis is the enrichment of pathways. [file 12967_2015_603_MOESM7_ESM.png]

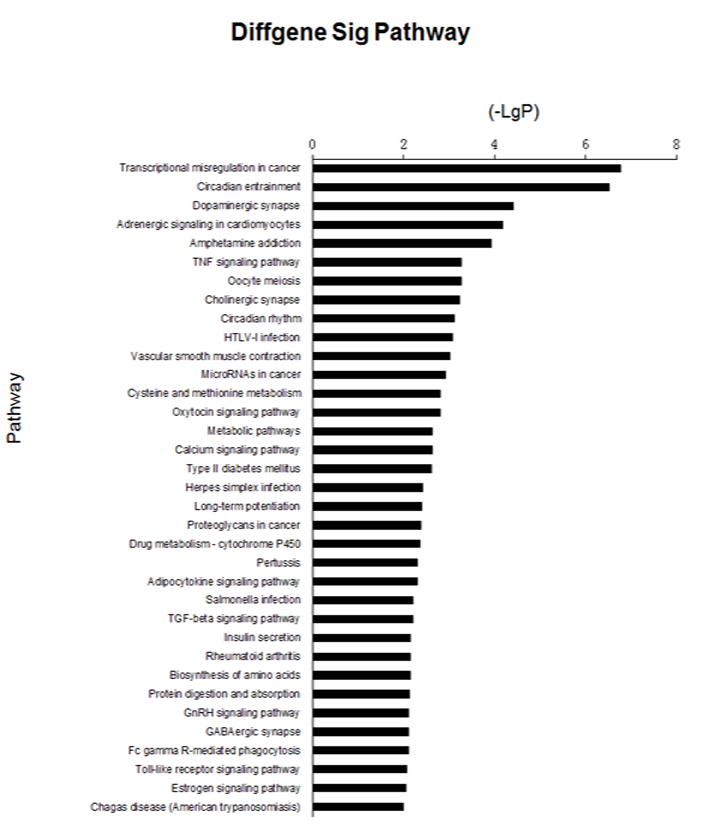

Supplement: Additional file 8: — Figure S2. Venn’s diagram for target genes of rno-miR-30a-5p, rno-miR-30c-5p, rno-miR-140-3p, rno-miR-143-3p, and rno-miR-145-5p predicted by MIRANDA, MICROCOSM, MIRDB. [file 12967_2015_603_MOESM8_ESM.tiff]

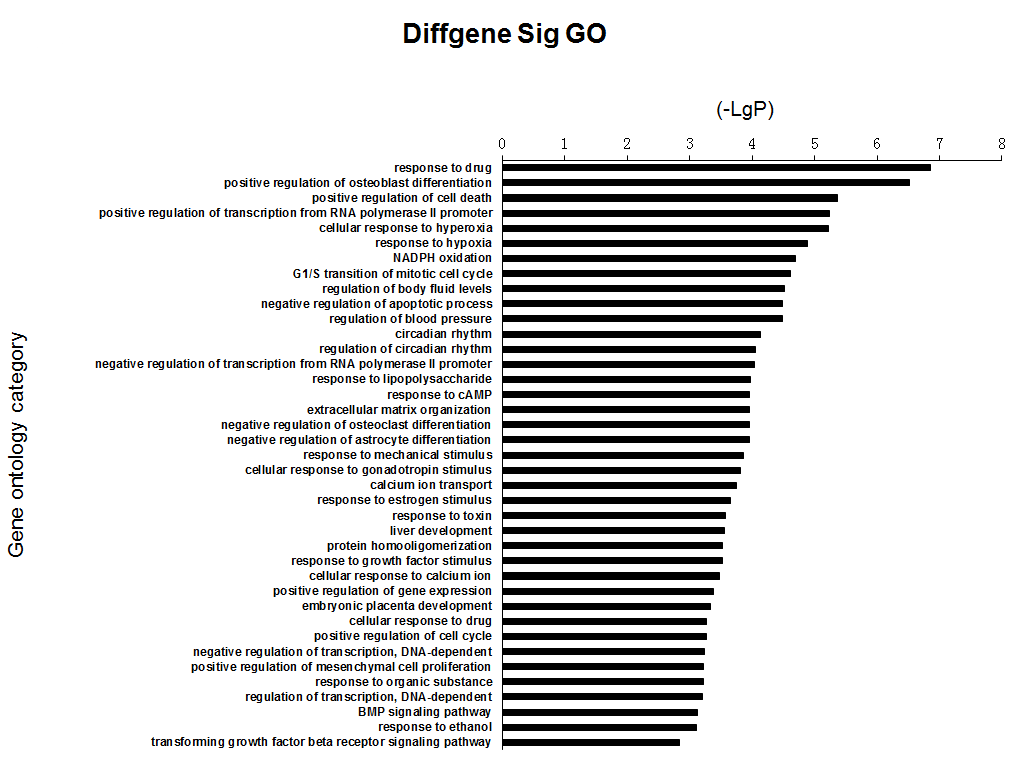

Supplement: Additional file 9: — Figure S3. GO-Analysis based on miRNA targeted genes showed significant function of target genes by rno-miR-30a-5p, rno-miR-30c-5p, rno-miR-140-3p, rno-miR-143-3p, and rno-miR-145-5p. The vertical axis is the significant functions, and the horizontal axis is the enrichment of functions. [file 12967_2015_603_MOESM9_ESM.tiff]

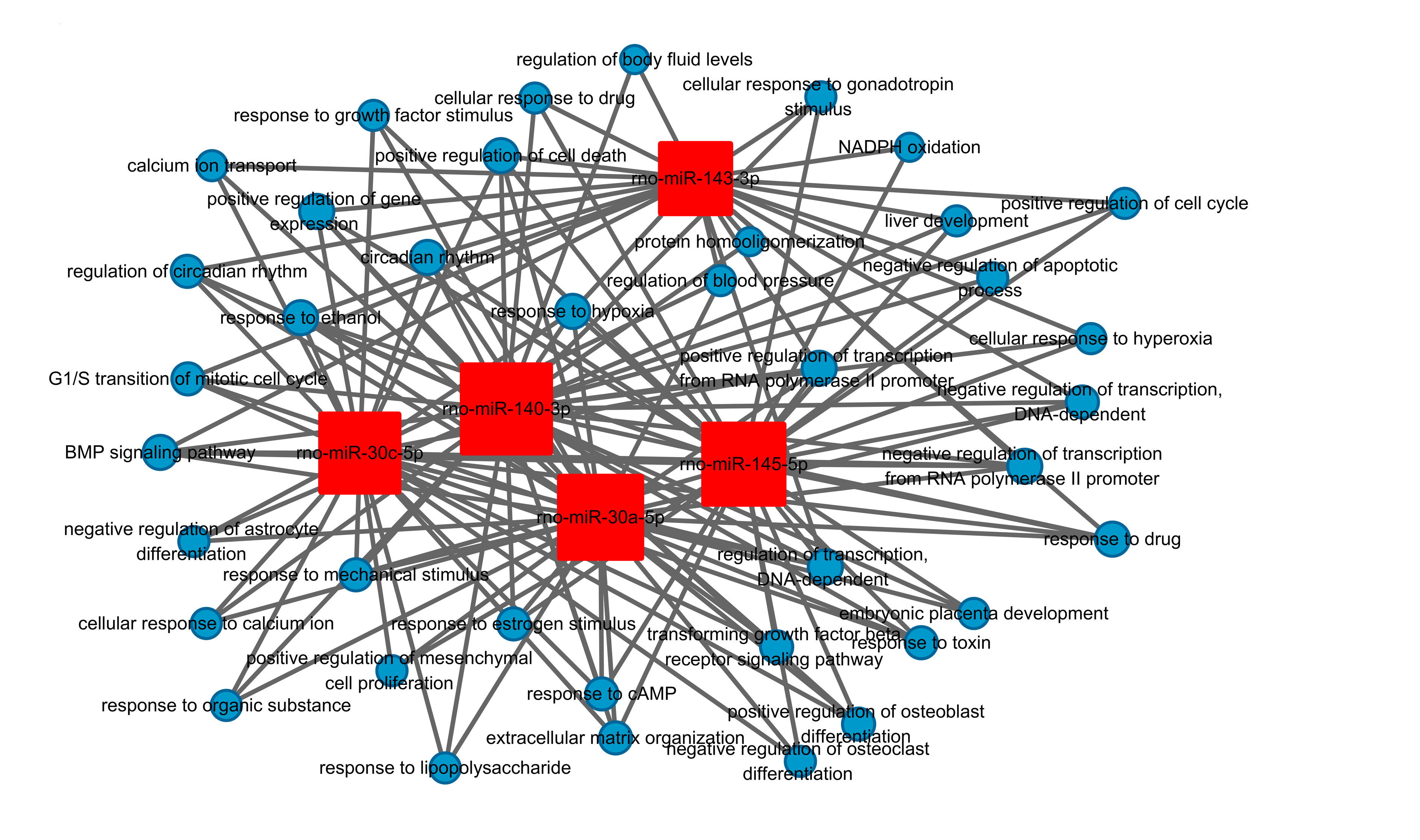

Supplement: Additional file 12: — Figure S4. MicroRNA-GO-Network, screening out the main function of the target genes regulated by rno-miR-30a-5p, rno-miR-30c-5p, rno-miR-140-3p, rno-miR-143-3p, and rno-miR-145-5p. The red nodes represent the microRNAs, and the blue nodes represent functions of the target genes. [file 12967_2015_603_MOESM12_ESM.tiff]

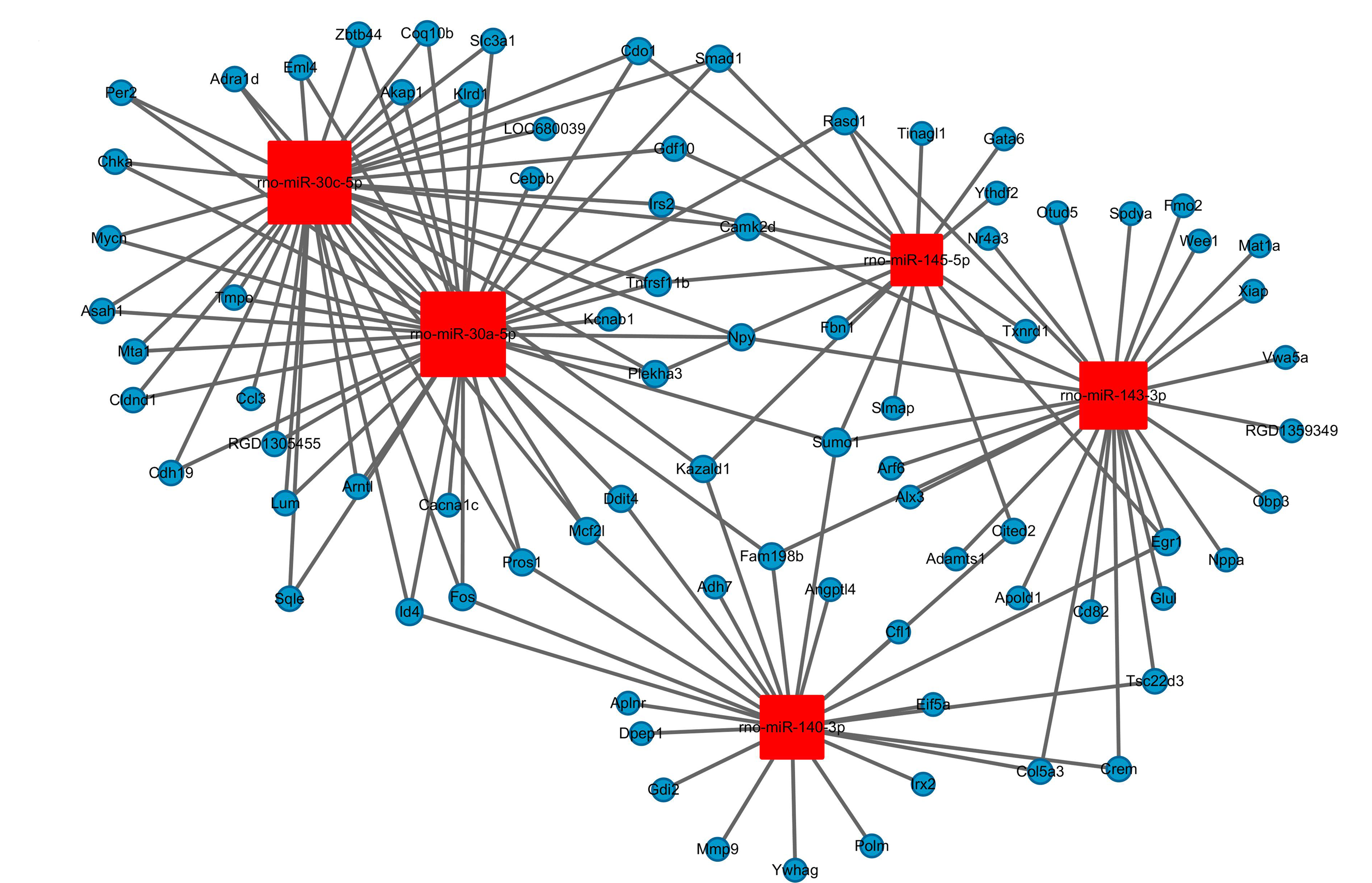

Supplement: Additional file 13: — Figure S5. Building the microRNA-Gene-Network by using the regulatory relationships between microRNAs and target genes. The red nodes represent the microRNAs, and the blue nodes represent the target genes. [file 12967_2015_603_MOESM13_ESM.tiff]
